# The risk of herpes zoster virus infection in patients with depression: A longitudinal follow-up study using a national sample cohort

**DOI:** 10.1097/MD.0000000000017430

**Published:** 2019-10-04

**Authors:** Hyo Geun Choi, Eui-Joong Kim, Young Kyung Lee, Miyoung Kim

**Affiliations:** a Department of Otorhinolaryngology-Head & Neck Surgery, Hallym University College of Medicine, Anyang; b Department of Psychiatry, Eulji University Eulji Hospital, Seoul; c Department of Laboratory Medicine, Hallym University College of Medicine, Anyang, Republic of Korea.

**Keywords:** depression, herpes zoster, Korea

## Abstract

The features of herpes zoster share some commonalities with depression, including decreased cellular immunity, a close correlation with nutritional status, and a higher prevalence in the elderly population. We aimed to assess the association between herpes zoster infection and depression in the Korean population.

We performed a longitudinal follow-up study of a nationwide sample cohort derived from the Korean National Health Insurance Service database. Individuals diagnosed with depression between 2002 and 2013 (n = 58,278) as well as matched controls (n = 233,112), with both groups comprising 34.3% male and 65.7% female subjects, were extracted and analyzed for the presence of herpes zoster infection. Depression was diagnosed based on the International Classification of Diseases tenth revision (ICD-10) codes F31–F39, while herpes zoster was diagnosed as ICD-10 B02.

The rate of herpes zoster infection was higher in the depressed group (6.8% [3967/58,278]) than in the control group (6.3% [14,689/233,122], *P* < .001). The adjusted hazard ratio (HR) for herpes zoster infection was 1.09 (95% CI: 1.05–1.13) in the depressed group (*P* < .001). Subgroup analyses revealed that the adjusted HRs for herpes zoster infection were higher only in women younger than 60 years among participants with depression. These HRs were 1.13 (95% CI: 1.02–1.25; *P* = .016) in women younger than 40 years and 1.11 (95% CI: 1.04–1.17; *P* < .001) in women aged 40–59 years.

Depression is a predictor of herpes zoster infection in Korean women younger than 60 years.

## 1 Introduction

Depression is characterized by a pervasive and persistent despondent mood, decreased confidence, and loss of interest in daily matters.^[1]^ Recent studies have suggested that depression will be one of the three leading types of disease burdens worldwide by 2030 along with ischemic heart disease (IHD) and HIV/AIDS.^[2,3]^ Therefore, knowledge of the physical comorbidities associated with depression is important for managing patients with this condition and preventing other physical illnesses. This is especially true since patients with depression often withdraw from societal interactions, which can cause physical symptoms to be easily missed.^[4]^

The immune system is integrated with other homeostatic mechanisms that are ultimately regulated by the brain.^[5,6]^ Additionally, there is a growing body of evidence that depression is not merely an emotional disorder but is also caused by disturbances in cellular immunity. A previous meta-analysis showed that depression was associated with reduced lymphocyte proliferation and a decrease in the proportions of lymphocytes and T cells, which are responsible for cell-mediated immunity.^[7]^ Other studies also found that depression was associated with dysregulated innate and adaptive cell-mediated immunity.^[8–10]^

Decreased cellular immunity is a main cause for higher susceptibility to infectious agents. As such, herpes zoster has been receiving particular attention in patients with depression. Commonly known as shingles, this infection is a painful neurocutaneous syndrome caused by the reactivation and replication of the varicella zoster virus (VZV).^[11]^ It is characterized by unilateral crops of painful and pruritic vesicles that appear in a dermatomal distribution.^[12–14]^ VZV establishes latency in the dorsal root ganglia, and its reactivation is associated with a decline in cell-mediated immunity as seen in patients with lymphoma and HIV infection, as well as those receiving chemotherapy and steroids.^[12–14]^ Herpes zoster shares some commonalities with depression, including decreased cellular immunity, a close association with nutrition, and a prevalence in the elderly population.^[12–15]^ Previous experimental studies have shown that VZV-specific responder cell frequency is markedly reduced in patients with depression.^[16]^ Additionally, depressed patients showed diminished VZV-specific cell-mediated immunity responses to zoster vaccines.^[11]^

To date, only a few studies have explored the risk of herpes zoster in patients with depression, even though many studies have hinted at the existence of such an association. Most case-control studies are from Western countries; hence, the data they produced might not be applicable to certain ethnic groups or individuals in other geographic locations. As such, little is known about the features of herpes zoster in Asian populations. To that end, we performed a prospective matched control follow-up study with a nationwide population-based dataset in South Korea to determine the risk of herpes zoster in patients with depression.

## 2 Materials and methods

### 2.1 Study population and data collection

The ethics committee of Hallym University approved the use of these data (approval number 2014-I148). The requirement for written informed consent was waived by the Institutional Review Board.

This national cohort study relied on data from the Korean Health Insurance Review and Assessment Service (HIRA)-National Sample Cohort. The Korean National Health Insurance Service, enrollment in which is mandatory, selects patients directly from a database that encompasses the entire Korean population (50 million individuals) to prevent non-sampling errors. Approximately 2% of the samples (1 million) were selected; these data were classified into 1476 levels (age [18 categories], sex [2 categories], and income level [41 categories]) using randomized stratified systematic sampling methods (via proportional allocation) to represent the entire population.^[17]^ The cohort database included

1. personal information,
2. health insurance claim codes (procedures and prescriptions),
3. diagnostic codes using the International Classification of Disease, tenth edition (ICD-10),
4. death records from the Korean National Statistical Office (using the Korean Standard Classification of Disease),
5. socio-economic data (residence and income), and
6. medical examination data for each participant collected between 2002 and 2013.

Because each Korean citizen is assigned a permanent 13-digit resident registration number, exact population statistics can be determined using this database. All Korean hospitals and clinics use this number to record individual patients in the medical insurance system; this avoids the risk of overlapping medical records even if a patient relocates to another geographical region. Moreover, all medical treatments in Korea can be tracked; moreover, a notice of death must legally be delivered to an administrative entity before a funeral can be held. Causes and dates of death are recorded by medical doctors on death certificates.

### 2.2 Participant selection

**Figure 1:**
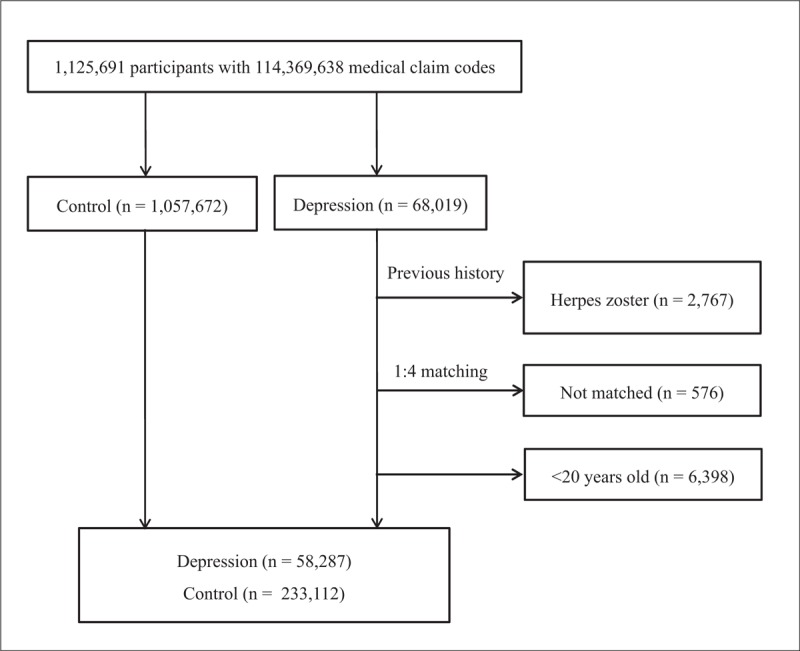
A schematic illustration of the participant selection process that was used in the present study. Of 1,125,691 participants, 58,287 individuals with depression were matched with 233,112 control participants according to age, group, sex, income group, region of residence, and past medical history.

From among 1,125,691 patients with 114,369,638 medical claim codes, we selected participants who were diagnosed with depression between 2002 and 2013 (Fig. 1). Depression was defined using the ICD-10 codes F31 (bipolar affective disorder) through F39 (unspecified mood disorder), as determined by a psychiatrist. From this population, we selected participants who were treated for depression ≥2 times (n = 68,019).

Herpes zoster was diagnosed as the ICD-10 code B02. From within the patient pool, we included only participants who were treated for herpes zoster at least twice or who were treated with antiviral medication at least once. A total of 64,152 patients diagnosed with herpes zoster between 2002 and 2013 were included.

Participants with depression were matched 1:4 with those in the control group that comprised individuals who were never diagnosed with depression within the same time period. The control group participants were selected from the remaining greater population (n = 1,057,672). The matches were processed for age, sex, income group, region of residence, and past medical history (including hypertension, diabetes, and dyslipidemia). To prevent selection bias, participants in the control group were each assigned a random number and were then selected in descending order. It was assumed that the control participants were involved at the same time as each matched depressed participant (index date); therefore, participants in the control group who died before the index date were excluded. Both the depression and control groups excluded participants who had histories of herpes zoster before the index date. As such, 2767 participants in the depression group were excluded, as were an additional 576 depressed participants for whom we could not identify matches as well as 6398 who were under 20 years of age. Ultimately, 1:4 matching resulted in the inclusion of 58,287 participants with depression and 233,112 control participants. Of note, the subjects were not matched for IHD; moreover, a history of cerebral stroke resulted in an increased number of dropouts when using our strict matching scheme owing to the lack of control participants.

### 2.3 Variables

Fourteen age groups were identified using 5-year intervals: 20–24, 25–29, 30–34…, and 85+ years. The income groups were initially divided into 41 classes (one health aid class, 20 self-employment health insurance classes, and 20 employment health insurance classes). These groups were recategorized into 11 classes (class 1 [lowest income] to 11 [highest income]). Regions of residence were divided into 16 areas according to administrative districts. These regions were regrouped into urban (Seoul, Busan, Daegu, Incheon, Gwangju, Daejeon, and Ulsan) and rural (Gyeonggi, Gangwon, Chungcheongbuk, Chungcheongnam, Jeollabuk, Jeollanam, Gyeongsangbuk, Gyeongsangnam, and Jeju) areas.

The medical histories of the participants were documented using ICD-10 codes. To ensure accurate diagnoses, the subjects were checked for the presence of hypertension (I10 and I15), diabetes (E10–E49), and dyslipidemia (E78) if they were treated ≥2 times. The presence of IHD (I24 and I25) and cerebral stroke (I60–I66) was checked if the participants were treated ≥1 time.

### 2.4 Statistical analyses

We used a Cox proportional hazards model to determine the hazard ratio (HR) for herpes zoster in individuals with depression. In this analysis, crude (simple) models as well as those adjusted for age, sex, income, region of residence, hypertension, diabetes, dyslipidemia, IHD, and cerebral stroke histories were used. The 95% confidence intervals (CIs) were calculated.

For the subgroup analyses, we divided the participants according to age (young [<40 years], middle aged [≥40 years and <60 years], and old [≥60 years]) as well as sex (men and women).

Two-tailed analyses were conducted, and *P* values less than .05 were considered significant. The results were statistically analyzed using SPSS v. 22.0 (IBM, Armonk, NY).

## 3 Results

The general characteristics of the control and depression groups are summarized in Table 1. The distributions of age, sex, income level, and region of residence were comparably matched between the 2 groups. The incidences of hypertension, diabetes, and dyslipidemia did not differ between the groups, while IHD and cerebral stroke were more prevalent in the depression group than in the control group (7.3% vs 5.6% [*P* < .001] for IHD and 13.4% vs 9.2% [*P* < .001] for cerebral stroke).

**Table 1:**
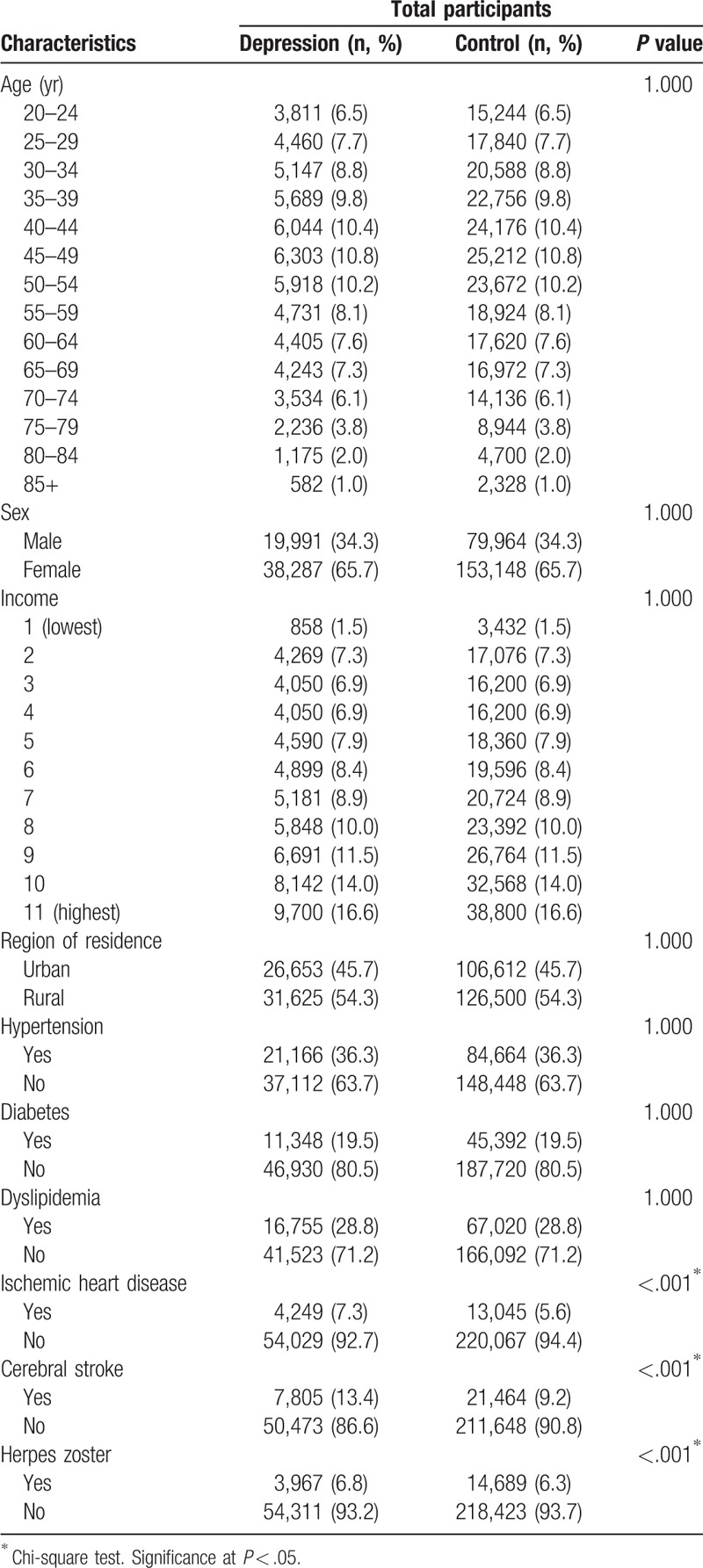
General characteristics of the participants.

Herpes zoster was more prevalent in the depression group than in the control group (6.8% [3,967/58,278] vs 6.3% [14,689/233,122], *P* < .001). The adjusted HR for herpes zoster in individuals with depression was 1.09 (95% CI: 1.05–1.13; *P* < .001); adjustment was for age, sex, income, region of residence, hypertension, diabetes, dyslipidemia, IHD, and cerebral stroke histories (Table 2).

**Table 2:**
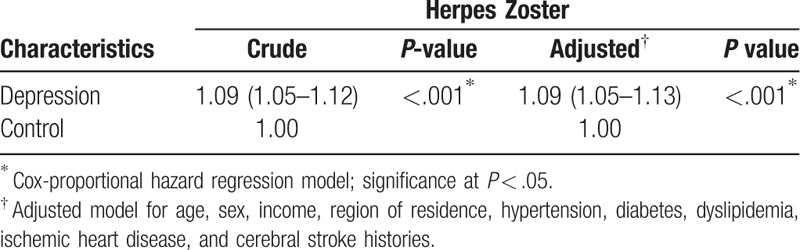
Crude and adjusted hazard ratios (95% confidence interval) of depression as associated with herpes zoster.

A subgroup analysis according to age and sex revealed that depression elevated the risk of herpes zoster only in women younger than 60 years (Table 3). The adjusted HR for herpes zoster in individuals with depression was 1.13 (95% CI: 1.02–1.25; *P* = .016) in women younger than 40 years (n = 62,585), and was 1.11 (95% CI: 1.04–1.17; *P* < .001) in women who were at least 40 years but younger than 60 years (n = 75,530). Men 60 years of age or older with depression showed an elevated risk of herpes zoster, with a crude HR of 1.11 (95% CI: 1.00–1.23; *P* < .046); however, the difference was no longer significant after adjusting for age; sex; income; region of residence; and histories of hypertension, diabetes, dyslipidemia, IHD, and cerebral stroke. The findings were not significant for any of the other depression subgroups.

**Table 3:**
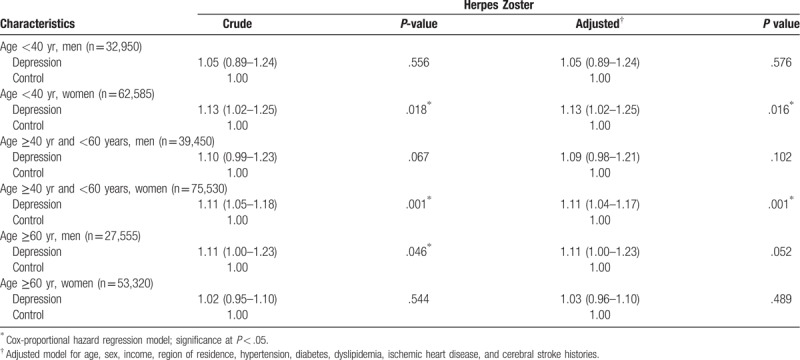
Subgroup analysis of crude and adjusted hazard ratios (95% confidence intervals) of the association between depression and herpes zoster.

## 4 Discussion

Our study revealed that the prevalence of herpes zoster was higher in the depression group than in the healthy control group (6.8% [3967/58,278] vs 6.3% [14,689/233,122], *P* < .001); the adjusted HR for depression among subjects with herpes zoster was 1.09 (95% CI: 1.05–1.13; *P* < .001). Subgroup analyses of subjects stratified by age and sex yielded the novel finding that the risk of herpes zoster was elevated only in women younger than 60 years.

The observation of an increased risk of herpes zoster in individuals with depression is consistent with the findings of previous studies, of which there are only a few. In a study of 22,886 Taiwanese subjects, Liao et al^[1]^ found a 1.3-fold higher incidence of herpes zoster in patients with depression than in controls (4.58 vs 3.54 per 1000 person-years), with an adjusted HR of 1.11 (95% CI: 1.01–1.21). In a meta-analysis of 3 studies that compared depressed patients to healthy controls in Western countries, Wang et al^[18]^ observed a slightly higher risk of VZV infection in the former (odds ratio = 2.07; CI 95%: 1.01–4.28; *P* = .048).

As mentioned in the Introduction section, the most probable explanation for this phenomenon is the decrease in cellular immunity in depressed patients. Maes^[19]^ described the influence of dysregulated cell-mediated immunity in depression, including increased serum levels of the soluble interleukin (IL)-2 receptor and the sCD8 molecule; increased numbers and percentages of T cells bearing T cell activation markers such as CD2+CD25+, CD3+CD25+, and HLA-DR+; increased stimulated production of interferon (IFN)γ; higher neopterin, which is also a marker for immune system activation and soluble tumor necrosis factor (TNF) receptor-1 or receptor-2 levels; induction of indoleamine 2,3-dioxygenase (IDO) with lowered levels of plasma tryptophan and increased levels of tryptophan catabolites along the IDO pathway (TRYCATs); and glucocorticoid resistance in immune cells. They also reported that anti-5-hydroxytryptamine (5-HT) antibody activity was significantly higher in depressed patients (54.1%) – particularly in those with melancholia (82.9%) – than in controls (5.7%). Patients with positive 5-HT antibodies showed increased serum neopterin and lysozyme, as well as plasma TNFα and IL-1; higher scores on the Hamilton Depression Rating Scale and the Fibromyalgia and Chronic Fatigue Syndrome Rating Scale; and more somatic symptoms including malaise and neurocognitive dysfunctions.^[20]^

In their review, Leonard and Maes^[21]^ suggested novel pathways (other than the above-described mechanisms) connecting cell-mediated immune activation to inflammation and depression, as follows:

1. induction of IDO by IFNγ and some pro-inflammatory cytokines is associated with depleted plasma tryptophan, which may interfere with brain 5-HT synthesis and contributes to increased production of anxiogenic and depressogenic tryptophan catabolites;
2. increased bacterial translocation may cause depression-like behaviors by activating the cytokine network, oxidative and nitrosative stress pathways, and IDO;
3. induction of the oxidative and nitrosative stress pathways causes damage to membrane ω3 polyunsaturated fatty acids (PUFAs), functional proteins, DNA, mitochondria, and autoimmune responses directed against intracellular molecules that may cause dysfunctions in intracellular signaling;
4. decreased levels of ω3 PUFAs and antioxidants, such as coenzyme Q10, glutathione peroxidase, or zinc, are associated with increased inflammatory potential, greater oxidative damage, onset of specific symptoms, and changes in the expression or functions of brain 5-HT and N-methyl-d-aspartate receptors.

Irwin et al^[16]^ showed that the VZV-specific responder cell frequency, which is a marker of VZV-specific cell-mediated immunity, is markedly lower in depressed patients than in control subjects, and is inversely correlated with the severity of depressive symptoms.^[22]^ In a subsequent study of 40 participants with major depressive disorder and 52 age-/sex-matched non-depressed controls, the authors reported that depressed patients showed diminished VZV-specific cell-mediated immunity responses to zoster vaccine.^[11]^ Additionally, they reported that treatment with antidepressant medication was associated with normalization of these responses. Separately, Liao et al^[1]^ suggested that nutritional deficiency could have contributed to the increasing rate of herpes zoster infection in depressive patients. Previous research linked depression to nutritional deficiencies that contribute to decreased immune response; the reactivation of VZV could be increased in patients with depression as a consequence of such deficiencies.^[23–27]^

Our study yielded the novel finding that the risk of herpes zoster was elevated only in women younger than 60 years in the subgroup analyses of subjects stratified by age and sex. Liao et al^[1]^ performed a similar subgroup analysis but showed that the risk of herpes zoster was increased in all subjects over the age of 35 years regardless of sex. Irwin et al^[11]^ demonstrated that the abovementioned VZV-specific immune responses were activated in reaction to the herpes zoster vaccine in women 60 years or older.

In a study whose design was the inverse of ours (exploring the incidence of depression in patients with herpes zoster), Chen et al^[28]^ showed that the diagnosis of herpes zoster at an older age (crude HR = 0.99, 95% CI: 0.98–1.00; adjusted HR = 0.99, 95% CI: 0.98–1.00) decreased the risk of developing major depression and any depressive disorder. They suggested that the development of depression and cell-mediated immunity dysfunction at an earlier age may lead to a greater vulnerability to herpes zoster infection. Alternatively, since the risks for both depression and herpes zoster increase as people age due to physical and lifestyle changes, which in turn lead to decreased immunity, increased comorbidities, and emotional incompetence, the increased risk of herpes zoster in elderly individuals with depression might have been counterbalanced (or perhaps under-evaluated) in the subgroup analysis. As such, further studies are required to elucidate the mechanisms underlying our finding.

Ours is the first study of its kind conducted among Koreans. Along with the study performed by Liao et al,^[1]^ our investigation revealed an increased risk of herpes zoster infection in a group of depressed individuals compared to healthy controls in an Asian population. We were not able to compare our results to those of studies previously performed in other ethnic populations because only a few such studies were designed comparably to ours; these were performed long ago and were based on relatively small sample sizes.^[18]^ Future studies performed in subjects of various ethnicities may both confirm an association between herpes zoster infection and antecedent depression, and also elucidate any underlying genetic factors that might contribute to the differences in these associations among different ethnicities.

The strength and reliability of this study are that it is based on a representative, large-scale sample from a cohort database consisting of 1 million subjects with a 12-year follow-up period. The size of the database has allowed us to analyze many more patients than required by the power calculation, and the sample size makes this the largest study of its kind to date. Additionally, as the dataset is based on claims registered in the HIRA (the nationwide compulsory health insurance system), this approach eliminated the risk of recall bias or missing data. We were able to choose matched controls while accounting for the potential confounding factors of age, sex, income group, and region of residence. Another strength of this study is the well-defined population; we included participants who were treated for depression ≥2 times or who were treated with antiviral medication ≥1 time owing to herpes zoster infection. Subjects with depression or herpes zoster received their diagnoses in either inpatient or outpatient clinics, which minimized the chance of selection bias that could arise from including only the most severely affected patients. The data on comorbidities among patients with depression rendered our cohort broadly representative. Moreover, the rates of associated chronic diseases, including IHD, in the depression and matched control groups were consistent with findings from previous epidemiologic studies conducted in South Korea.^[29–31]^ Therefore, our finding of an increased incidence of herpes zoster in subjects with depression in the Korean population is robust and representative.

A limitation of our study is that we could not adjust the data for some factors that may have affected the prevalence of depression, including obesity, smoking, alcohol consumption, and dietary habits. Nevertheless, such factors are not considered crucial for the development of depressive disorders; thus, we assume that our findings would not have been very different even if these factors had been taken into account. The other potential confounder regarding our results could be the actual chronology of the development of depression and herpes zoster infection, that is, whether depression truly preceded herpes zoster in our study subjects. To ensure that the development of depression was followed by herpes zoster infection, we excluded participants who had a history of herpes zoster before the index date. Nevertheless, the possibility of preexisting subclinical herpes zoster infection was not completely ruled out by our method.

In conclusion, our longitudinal follow-up study using a national sample cohort showed that the risk of herpes zoster is elevated only in women with depression who are younger than 60 years of age.

## Author contributions

**Conceptualization:** Eui-Joong Kim, Young Kyung Lee, Miyoung Kim.

**Data curation:** Hyo Geun Choi, Eui-Joong Kim, Young Kyung Lee.

**Formal analysis:** Eui-Joong Kim, Young Kyung Lee.

**Investigation:** Hyo Geun Choi, Eui-Joong Kim, Young Kyung Lee.

**Supervision:** Miyoung Kim.

**Writing – original draft:** Hyo Geun Choi.
